# Spike Density Quantitative Trait Loci Detection and Analysis in Tetraploid and Hexaploid Wheat Recombinant Inbred Line Populations

**DOI:** 10.3389/fpls.2021.796397

**Published:** 2021-12-16

**Authors:** Jianing You, Hang Liu, Surong Wang, Wei Luo, Lulu Gou, Huaping Tang, Yang Mu, Mei Deng, Qiantao Jiang, Guoyue Chen, Pengfei Qi, Yuanying Peng, Liwei Tang, Ahsan Habib, Yuming Wei, Youliang Zheng, Xiujin Lan, Jian Ma

**Affiliations:** ^1^Triticeae Research Institute, Sichuan Agricultural University, Chengdu, China; ^2^Panzhihua Academy of Agricultural and Forestry Sciences, Panzhihua, China; ^3^Biotechnology and Genetic Engineering Discipline, Khulna University, Khulna, Bangladesh

**Keywords:** spike density, quantitative trait loci, wheat, wheat55K SNP array, pyramiding analysis

## Abstract

Spike density (SD) is an agronomically important character in wheat. In addition, an optimized spike structure is a key basis for high yields. Identification of quantitative trait loci (QTL) for SD has provided a genetic basis for constructing ideal spike morphologies in wheat. In this study, two recombinant inbred line (RIL) populations (tetraploid RIL AM and hexaploid RIL 20828/SY95-71 (2SY)) previously genotyped using the wheat55K SNP array were used to identify SD QTL. A total of 18 QTL were detected, and three were major and one was stably expressed (*QSd.sau-2SY-7A.2, QSd.sau-AM-5A.2*, *QSd.sau-AM-7B*, and *QSd.sau-2SY-2D*). They can explain up to 23.14, 19.97, 12.00, and 9.44% of phenotypic variation, respectively. QTL × environment and epistatic interactions for SD were further analyzed. In addition, pyramiding analysis further revealed that there were additive effects between *QSd.sau-2SY-2D* and *QSd.sau-2SY-7A.2* in 2SY, and *QSd.sau-AM-5A.2* and *QSd.sau-AM-7B* in AM. Pearson’s correlation between SD and other agronomic traits, and effects of major or stable QTL on yield related traits indicated SD significantly impacted spike length (SL), spikelet number per spike (SNS) and kernel length (KL). Several genes related to spike development within the physical intervals of major or stable QTL were predicted and discussed. Collectively, our research identified QTL with potential applications for modern wheat breeding and broadening the genetic basis of SD.

## Introduction

As one of the most important food crops in the world, the yield of common wheat (*Triticum aestivum* L.) should be increased to meet the growing demand for food for human beings (Zhou et al., 2017; Xu et al., 2018). The spike is an important part of the wheat plant. Cultivating wheat varieties with longer spike length (SL) and higher spike density (SD) could increase yield (Faris et al., 2014; Li et al., 2016). Thus, as a spike trait controlled by genes and influenced by the environment (Ma et al., 2007), identification of quantitative trait loci (QTL) associated with SD has importantly theoretical value for breeding high-yield wheat varieties.

*Q*, *Compactum* (*C*), and *Sphaerococcum* (*S1*) are three well-known genes related to spike development in common wheat (Fellers et al., 2003). The *Q* gene, located on the long arm of chromosome 5A, not only plays a role in spike morphogenesis, but also has pleiotropic effects on seed threshability, spike emergence time, and plant height (PH) (Faris and Gill, 2002; Fellers et al., 2003; Simons et al., 2006; Xu et al., 2018). The *C* gene, located on chromosomes 2D near the centromere, is involved in regulating SD, grain shape, and grain number per spike (Johnson et al., 2008). The *S1* gene on chromosome 3D defines grain shape and SD in wheat (Prabhakararao, 1977). However, *C* or *S* genes do not exist in tetraploid cultivars or varieties since they do not possess D-genome chromosomes. Thus, variation in spike morphology of tetraploid wheat may be caused by genes other than *Q*, *C*, or *S* or by alleles of these three genes on homologous chromosomes (Faris et al., 2014; Zhou et al., 2017). Therefore, it is necessary to excavate more QTL or genes associated with SD in tetraploid wheat.

Previous studies have reported that dwarf genes were involved in the regulation of wheat spike development. For example, *Rht-9* and *Rht-12* are gibberellin-sensitive genes, and they can affect heading date (Ellis et al., 2005); *Rht-8* was close to the marker *Xgwm261* (Korzun et al., 1998), while a QTL for SD was also reported to be tightly linked to this marker (Heidari et al., 2011; Zhao et al., 2013), indicating that dwarf genes may have some intrinsic interaction with SD. Additionally, photoperiod (*Ppd*), vernalization (*Vrn*) as well as earliness *per se* (*Eps*) genes also have certain effects on spike development (Alvarez et al., 2016; Guedira et al., 2016).

In order to further excavate major loci associated with spike development among modern wheat varieties, scholars worldwide have identified a large number of QTL for spike traits (Jantasuriyarat et al., 2004; Kumar et al., 2007; Ma et al., 2007; Cui et al., 2012; Zhai et al., 2016; Tao et al., 2019; Kuang et al., 2020; Li et al., 2021). For example, Ma et al. (2007) analyzed five spike traits in a recombinant inbred line (RIL) population and an immortalized F_2_ population. They found QTL controlling SD were distributed on chromosomes 1A, 4A, 5A, 5B, 2D, and 7D, and single QTL was able to explain 7.9–36.3% of phenotypic variation. Based on the genetic map constructed using the wheat55K SNP array, Liu et al. (2019) detected 24 SD QTL. Three of them were major QTL being located on chromosomes 2D, 4B, and 5B, and stably expressed in various environments, indicating that high-density genetic mapping is a critical approach to QTL mapping. Although loci associated with spike development in common wheat have been extensively studied, there have been few studies on identification of loci in tetraploid wheat (*Triticum turgidum* L.). There are still many loci that could be mined and utilized from such germplasm resources.

In the present study, two RIL populations previously genotyped using the wheat55K SNP array were used to identify SD QTL in combination with the phenotypic data from multiple environments. The correlations between SD and other agronomic traits were analyzed. Major SD QTL were identified. Pyramiding analysis for these major QTL was performed. In addition, candidate genes for QTL were also predicted.

## Materials and Methods

### Plant Materials

Three RIL populations of wheat were used in the study: hexaploid population 20828/SY95-71 (2SY, 128 F_7_ RILs including parents) (Liu et al., 2020), hexaploid population 20828/Chuanmai60 (2CM, 207 F_2:3_ lines) (Ma et al., 2019b), and tetraploid population Ailanmai (AL)/LM001 (AM, 121 F_8_ RILs including parents) (Mo et al., 2021).

The wheat line 20828 is highly resistant to stripe rust disease (Ma et al., 2019b), and has a short spike extension length (Li et al., 2020), a large uppermost-internode diameter (Liu et al., 2021), and multiple spikelets per spike (Ding et al., 2021). SY95-71 is a stable line with a well-developed root system (Zheng et al., 2019) and a relatively high number of tillers (Liu et al., 2020). Chuanmai60 is a commercial cultivar. AL is a unique germplasm resource from China, and has characteristics of dwarf plants and multiple florets (Liu et al., 1999). As a wild emmer wheat line, LM001 exhibits fewer kernels per spikelet, non-free threshability and long awns (Mo et al., 2021). The 2SY and AM populations were used for QTL identification, and the 2CM population was used for verification of major QTL identified in the 2SY population.

### Phenotypic Evaluation

Three populations and their parental lines were evaluated at Wenjiang (WJ, 103° 51′ E, 30° 43′ N), Chongzhou (CZ, 103° 38′ E, 30° 32′ N), Ya’an (YA, 103° 0′ E, 29° 58′ N) in China, and Khulna (KB, 89° 34′ E, 22° 49′ N) in Bangladesh during 2017–2021.

The 2SY population was planted in seven environments, encoded as 2017WJ, 2018WJ, 2017CZ, 2018CZ, 2017YA, 2018YA, and 2018KB, respectively, based on the year and location. The AM population was planted in eight environments: 2017CZ, 2018CZ, 2019CZ, 2020CZ, 2021CZ, 2020WJ, 2021WJ, and 2020YA. The validation population 2CM was planted in 2018CZ. The RILs and their parents were planted in a single row for each environment. Each line consisted of 15 seeds evenly planted in a single 1.5-m row with 0.3 m between rows. Field management was conducted in accordance with the general practice of wheat production.

In the study, three individual plants with consistent growth of each line were selected to measure agronomic traits in the 2SY population and five individual plants with consistent growth of each line were selected to measure agronomic traits in the AM population. The phenotypic data of agronomic traits used in this experiment have been measured in previous studies, including spikelet number per spike (SNS), SL, PH, anthesis date (AD), productive tiller number (PTN), thousand kernel weight (TKW), kernel length (KL), kernel number per spike (KNS), kernel number per spikelet (KNL), and kernel width (KW). The agronomic traits of the 2SY populations were measured by Liu et al. (2020) (PH, AD, TKW, PTN, SNS), Ding et al. (2021) (SNS), Li et al. (2020) (SL), and Qu et al. (2021) (KL, KW). The phenotype values for SNS in 2018KB were determined by Ding et al. (2021), and the SNS data across remainder environments was determined by Liu et al. (2020). The agronomic traits of the AM population were measured by Mo et al. (2021) (PH, AD, TKW, SL, SNS, PTN, KNL and KNS) and Zhou et al. (2021) (KL, KW). The phenotypic data of SL and SNS in the 2CM population were measured by Ma et al. (2019a). Furthermore, SD was obtained by dividing SNS by SL. The detailed information of agronomic traits in different environments are presented in Supplementary Table 1.

### Data Analysis

The best linear unbiased prediction (BLUP) of agronomic traits and the broad-sense heritability (*H*^2^) of SD were calculated using SAS version 9.1 (SAS Institute, Cary, NC, United States). Based on phenotypic data and BLUP values, IBM SPSS 27 (IBM SPSS, Armonk, NY, United States) was used for Pearson’s correlation analysis to assess the relationships between SD and agronomic traits. Significant differences were evaluated using Student’s *t*-test. Origin 2018^1^ was used to describe the frequency distribution for phenotypic data from the two populations.

### Quantitative Trait Loci Mapping

Two genetic linkage maps constructed based on the wheat55K SNP array were used in the present study. The genetic map of the 2SY population covered a total genetic distance of 4,273.03 cM containing 2529 bin markers, and the mean interval between markers was 1.69 cM (Liu et al., 2020). In the AM population, the genetic distance for linkage maps was 2411.8 cM containing 1150 bin markers, and the mean interval between markers was 2.10 cM (Mo et al., 2021).

Individual environment QTL detection was performed using the biparental populations (BIP) module with inclusive composite interval mapping (ICIM) in IciMaing4.1. To improve the reliability of QTL results, the step was set to 1 cM, the PIN value was 0.001, and the logarithm of odds (LOD) score threshold was set to 3. Then, the multi-environment trials (MET-ADD) model in IciMapping4.1 was used to analyze the interaction between QTL and environment (Step = 1 cM, PIN = 0.001, and LOD = 7), and the epistatic effects between QTL were analyzed by multi-environmental trials (MET-EPI) in IciMapping4.1. In this study, QTL identified in two or more environments were treated as stable, and those explained more than 10% of phenotypic variation explained (PVE) were considered major loci. QTL were named according to the Catalogue of Gene Symbols for Wheat (McIntosh et al., 2013), where “sau” represents “Sichuan Agricultural University”, 2SY and AM represent population names.

### Physical Intervals of the Quantitative Trait Loci and Comparison With Previously Reported Quantitative Trait Loci

Sequences of flanking markers for a given QTL were blasted against the genomes of “Chinese spring” (CS; v2.1) (Zhu et al., 2021), wild emmer (Zavitan; v2.0) (Zhu et al., 2019), and *Aegilops tauschii* (Aet; v4.0) (Luo et al., 2017) to determine the corresponding physical intervals. QTL were determined to check if they were novel loci or not by comparing their physical locations with those of reported ones. Furthermore, candidate genes with functional annotations were obtained from the Triticeae Multi-omics Center^2^ and UniProt^3^.

## Results

### Phenotype Analysis

**FIGURE 1:**
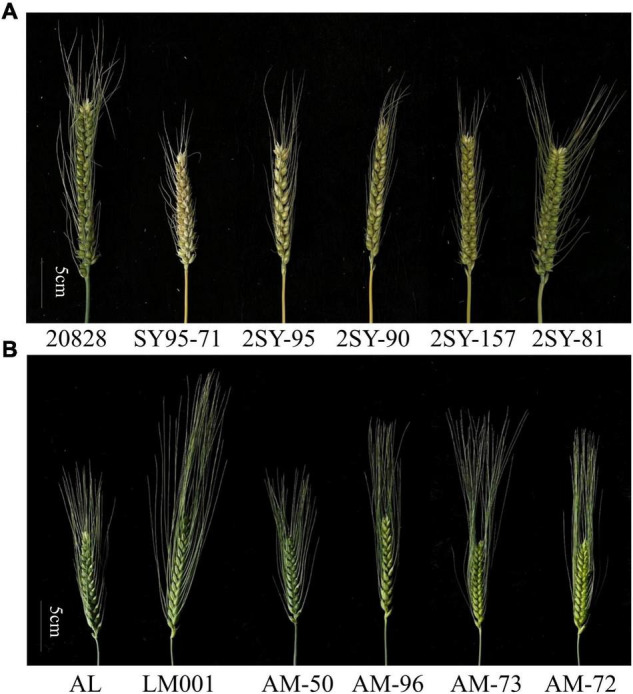
Spike morphology of 20828, SY95-71, and four selected lines **(A)** and AL, LM001 and four selected lines **(B)** (scale bar = 5 cm).

Significant differences between the parents of the 2SY and AM populations were observed in several environments (Figure 1). The phenotypic values of 2SY and AM RIL populations and their corresponding parents were statistically analyzed under multiple environments and based on BLUP datasets (Table 1).

**TABLE 1:** Phenotypic evaluation of spike density (SD) for the parents and two recombinant inbred lines (RIL) populations in different environments.

Population	Environment	Mean of female parent (20828 or Ailanmai)	Mean of male parent (SY95-71 or LM001)	Min–Max	Mean	STD	*Skew*	*Kurt*	*H* ^2^
2SY	2017WJ	1.71**	2.04	1.34–2.84	1.86	0.27	1.31	2.85	
	2017CZ	1.75	1.94	1.34–3.19	1.84	0.30	0.98	1.12	
	2017YA	2.06	2.24	1.58–3.35	2.19	0.35	1.19	3.10	
	2018WJ	2.05*	2.26	1.48–3.33	2.10	0.33	0.87	0.84	
	2018CZ	2.13	2.30	1.60–3.25	2.17	0.32	0.96	1.55	
	2018YA	2.49	2.66	1.57–3.55	2.38	0.39	0.84	0.48	
	2018KB	2.08**	2.74	1.35–4.14	2.41	0.58	0.65	0.57	
	BLUP	1.95	2.30	1.70–2.80	2.14	0.21	0.82	0.90	0.67
AM	2017CZ	2.06	2.02	1.66–2.73	2.08	0.20	0.62	0.87	
	2018CZ	2.40	2.44	1.88–3.03	2.29	0.21	0.57	0.95	
	2019CZ	2.30	2.09	1.65–2.67	2.09	0.20	0.58	0.37	
	2020CZ	2.61	2.65	2.04–3.48	2.50	0.25	0.79	1.69	
	2020WJ	2.55*	2.06	1.72–3.31	2.27	0.25	0.63	1.23	
	2020YA	2.41	2.41	1.84–3.85	2.70	0.38	0.56	0.57	
	2021CZ	2.30	2.12	1.90–3.13	2.30	0.21	0.84	1.21	
	2021WJ	2.29*	2.09	1.68–2.71	2.20	0.20	0.54	0.06	
	BLUP	2.01	2.87	2.01–2.87	2.31	0.15	0.64	1.03	0.69

2SY, 20828/SY95-71; AM, AL/LM001; WJ, Wenjiang; CZ, Chongzhou; YA, Ya’an; KB, Khulna, in Bangladesh; BLUP, best linear unbiased prediction environments; STD, standard deviation; Skew, skewness; Kurt, kurtosis; H^2^, the broad-sense heritability. * Significance level at P < 0.05; ** Significance level at P < 0.01.

**FIGURE 2:**
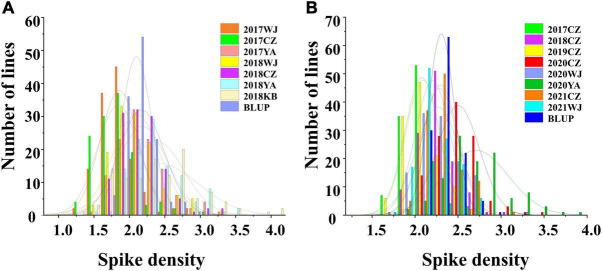
The phenotype and frequency distribution of spike density (SD) in the 2SY population **(A)** and AM population **(B)** under different environments.

In the 2SY RILs, variance analysis results showed that the SD of SY95-71 was significantly higher than that of 20828 in 2017WJ, 2018WJ, and 2018KB (*P* < 0.05), SD showed prominent variation, varying between 1.34 and 4.14. AL SD was significantly higher than LM001 in 2020WJ and 2021WJ environments (*P* < 0.05), while there was no significant difference in other environments. And a SD range from 1.65 to 3.85 was observed in AM RILs. The frequency distribution presented an approximately normal distribution and was bidirectionally transgressive in two RIL populations (Figure 2). The *H*^2^ in the 2SY and AM populations were 0.67 and 0.69, respectively. Moreover, significant correlations for SD among different environments in the 2SY and AM populations were detected (Supplementary Table 2). The SD phenotypes in the 2SY population exhibited significant correlations in all environments except 2018KB (0.48 ≤ *r* ≤ 0.79). The values of SD in the AM population were significantly correlated among all environments (0.24 ≤ *r* ≤ 0.61).

### Correlations Between Spike Density and Other Agronomic Traits

Correlation analysis between SD and other agronomic traits was conducted based on BLUP values (Table 2). In the 2SY and AM populations, SD was negatively correlated with KL and SL, but positively correlated with SNS (*P* < 0.01). Moreover, TKW was significantly and negatively correlated with SD in the 2SY population (*P* < 0.01).

**TABLE 2:** Correlation analysis between SD and other agronomic traits in two recombinant inbred lines (RIL) populations.

Traits	PH	AD	SL	SNS	TKW	PTN	KL	KW
2SY-SD	−0.07	−0.04	−0.60**	0.43**	−0.35**	−0.03	−0.41**	−0.12
AM-SD	−0.02	0.13	−0.45**	0.32**	0.15	0.03	−0.24**	−0.16

2SY, 20828/SY95-71; AM, AL/LM001; SD spike density; PH, plant height; AD, anthesis date; SL, spike length; SNS, spikelet number per spike; TKW, thousand kernel weight; PTN, productive tiller number; KL, kernels length; KW, kernels width. ** Significance level at P < 0.01.

### Quantitative Trait Loci Mapping of Spike Density and Prediction of Candidate Genes

**FIGURE 3:**
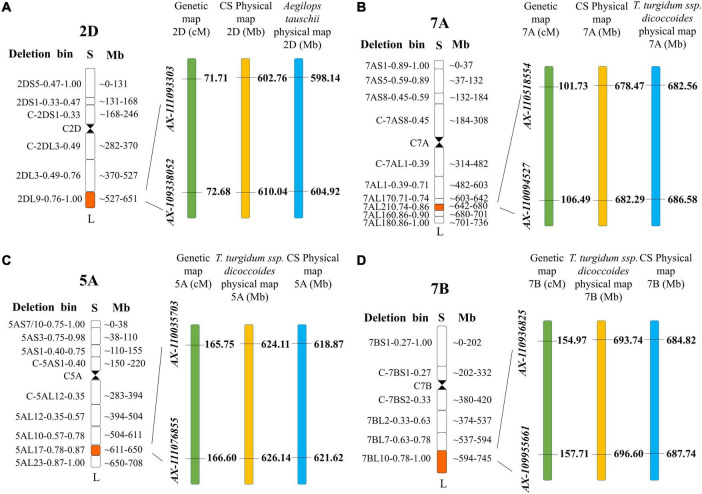
The maps of major QTL. **(A)** QSd.sau-2SY-2D; **(B)** QSd.sau-2SY-7A.2; **(C)** QSd.sau-AM-5A; **(D)** QSd.sau-AM-7B.

**FIGURE 4:**
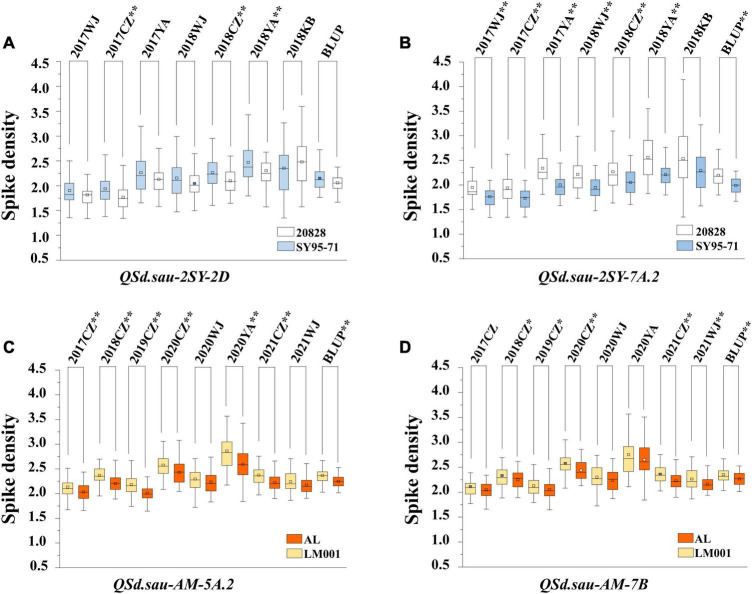
The effect of major QTL *QSd.sau-2SY-2D* **(A)**, *QSd.sau-2SY-7A.2* **(B)**, *QSd.sau-AM-5A.2* **(C)**, and *QSd.sau-7B* **(D)**. 20828 and SY95-71 indicate the phenotypes of the 2SY population with and without positive alleles of the corresponding QTL, respectively; AL and LM001 indicate the phenotypes of the AM population with and without positive alleles of the corresponding QTL, respectively. * Significance level at *P* < 0.05; ** Significance level at *P* < 0.01.

In total, 18 QTL for SD were identified in the two RIL populations by single-environment analysis, and they were distributed on chromosomes 1B, 2A, 2D, 3A, 3B, 4A, 4B, 5A, 5B, 6D, 7A, and 7B. A single QTL was able to explain 4.16–23.14% of the phenotypic variation (Table 3). Three QTL were regarded as major (including *QSd.sau-2SY-7A.2, QSd.sau-AM-5A.2*, and *QSd.sau-AM-7B*) and one can be stably expressed in multiple environments (*QSd.sau-2SY-2D*). Due to the stability of the *QSd.sau-2SY-2D*, it was further analyzed together with the major ones in the present study. In the 2SY population, *QSd.sau-2SY-2D* was stably expressed in three environments and the BLUP dataset, and it was mapped to the interval *AX-111093303∼AX-109338052*, and explained 4.45–9.44% of the phenotypic variance. The positive allele at this locus was from SY95-71. The stably expressed locus *QSd.sau-2SY-2D* was physically located at 602.76–610.04 Mb on 2D of the CS genome and 598.14–604.92 Mb on 2D of the *A. tauschii* genome, respectively (Figure 3A). According to the flanking markers of *QSd.sau-2SY-2D*, 2SY RILs could be divided into two groups (with or without the positive allele of *QSd.sau-2SY-2D*). The phenotypic values for SD carrying positive alleles were significantly higher than those with negative ones (*P* < 0.05) (Figure 4A).

**TABLE 3:** Quantitative trait loci (QTL) for spikelet density (SD) in two recombinant inbred lines (RIL) populations under different environments.

Populations	QTL	Environments	Position (cM)	Left marker	Right marker	LOD	PVE (%)	Add
2SY	*QSd.sau-2SY-2A*	2018WJ	41.97–43.47	*AX-109397555*	*AX-111595047*	3.11	9.16	0.11
	*QSd.sau-2SY-2D*	2017CZ	71.71–72.68	*AX-111093303*	*AX-109338052*	3.21	9.14	–0.09
		2018CZ	71.71–72.68	*AX-111093303*	*AX-109338052*	4.81	4.45	–0.10
		2018YA	71.71–72.68	*AX-111093303*	*AX-109338052*	3.78	9.44	–0.13
		BLUP	71.71–72.68	*AX-111093303*	*AX-109338052*	3.36	7.33	–0.06
	*QSd.sau-2SY-4A*	2018CZ	81.51–82.33	*AX-111045592*	*AX-108734258*	12.63	14.19	–0.17
	*QSd.sau-2SY-4B*	2017WJ	39.27–44.89	*AX-110033929*	*AX-108935259*	3.50	7.79	–0.08
	*QSd.sau-2SY-5A*	2018CZ	56.91–57.64	*AX-109362376*	*AX-108878364*	4.38	4.16	–0.09
	*QSd.sau-2SY-6D*	2018CZ	169.59–170.43	*AX-110412658*	*AX-109195537*	5.95	5.90	–0.11
	*QSd.sau-2SY-7A.1*	2017CZ	77.29–86.69	*AX-111511322*	*AX-110483331*	4.58	14.93	0.11
	*QSd.sau-2SY-7A.2*	2017WJ	101.73–103.88	*AX-110518554*	*AX-110442528*	5.32	12.51	0.10
		2017YA	103.88–106.49	*AX-110442528*	*AX-110094527*	8.09	21.10	0.18
		2018WJ	101.73–103.88	*AX-110518554*	*AX-110442528*	7.58	16.69	0.15
		2018CZ	101.73–103.88	*AX-110518554*	*AX-110442528*	10.12	10.56	0.15
		2018YA	101.73–103.88	*AX-110518554*	*AX-110442528*	8.30	23.14	0.21
		BLUP	100.63–101.73	*AX-108735843*	*AX-110518554*	9.60	23.44	0.11
	*QSd.sau-2SY-7A.3*	2018WJ	202.86–204.53	*AX-109947373*	*AX-111044435*	3.66	7.44	–0.10
AM	*QSd.sau-AM-3A*	2020CZ	51.33–53.05	*AX-109316902*	*AX-110445703*	3.28	7.29	0.08
	*QSd.sau-AM-5A.1*	2019CZ	156.06–162.34	*AX-110021952*	*AX-110503841*	4.81	16.77	–0.08
	*QSd.sau-AM-5A.2*	2018CZ	165.75–166.60	*AX-110035703*	*AX-111076855*	6.25	19.97	–0.09
		2020YA	165.75–166.60	*AX-110035703*	*AX-111076855*	3.02	12.29	–0.13
		BLUP	165.75–166.60	*AX-110035703*	*AX-111076855*	48.58	15.11	–0.29
	*QSd.sau-AM-7A.1*	2021WJ	76.99–85.22	*AX-111699124*	*AX-108740541*	3.16	9.89	0.06
	*QSd.sau-AM-7A.2*	2020CZ	162.62–107.05	*AX-109860028*	*AX-109624261*	3.82	8.59	0.08
	*QSd.sau-AM-1B*	2018CZ	24.81–27.15	*AX-110430183*	*AX-110539078*	3.60	10.86	0.07
	*QSd.sau-AM-3B*	2021CZ	48.34–49.69	*AX-109393346*	*AX-110548993*	4.51	13.47	0.08
	*QSd.sau-AM-5B*	2021WJ	40.88–41.31	*AX-109330727*	*AX-111107210*	6.14	20.23	0.09
	*QSd.sau-AM-7B*	2020CZ	154.97–157.71	*AX-110936825*	*AX-109955661*	4.57	11.06	–0.10
		2021CZ	154.97–157.71	*AX-110936825*	*AX-109955661*	4.07	12.00	–0.07

*PVE, phenotype variance explained; LOD, logarithm of odds; Add, additive effect of a QTL; BLUP, phenotype values based on the best linear unbiased prediction.*

*QSd.sau-2SY-7A.2* was identified under five environments as well as in the BLUP dataset, and its positive allele was from 20828. This QTL was able to explain 10.56–23.44% of the phenotypic variance, and its LOD value ranged from 5.32 to 10.12. *AX-110518554* and *AX-110094527* were its flanking markers and it was located at 678.47–682.29 and 682.56–686.58 Mb on chromosome 7A of the CS and *A. tauschii* genome, respectively (Figure 3B). We further divided the 2SY population into two groups, one with alleles from SY95-71 and one with alleles from 20828. Lines with 20828 alleles had larger SD values than those with SY95-71 alleles in all environments except 2018KB (*P* < 0.01) (Figure 4B).

In the AM population, *QSd.sau-AM-5A.2* was identified in a 0.85 cM region between markers *AX-110035703* and *AX-111076855* under three environments and the BLUP dataset. It explained 12.29–19.97% of phenotypic variance, and its LOD value was up to 48.58. Then, this locus was anchored at 618.87–621.62 and 684.82–687.74 Mb on chromosome 5A of the CS and wild emmer reference genome, respectively (Figure 3C).

*QSd.sau-AM-7B*, with a LOD value ranging between 4.07 and 4.57, was detected in two environments, and it accounted for 11.06–12.00% of the phenotypic variance. The positive alleles of *QSd.sau-AM-5A.2* and *QSd.sau-AM-7B* were both contributed by LM001. The AM population was grouped and compared using the flanking markers of these two QTL (Figures 4C,D). Compared to the RILs with alleles from AL, those with LM001 alleles showed a significant increase in SD across multiple environments. Moreover, the physical position of *QSd.sau-AM-7B* was at 684.82–687.74 and 693.74–696.60 Mb on 7B of the CS and wild emmer reference genome, respectively (Figure 3D). Further, candidate genes were analyzed in the regions with these major and stable QTL. Based on the reference genome of CS v2.1 and *A. tauschii*, 47 orthologs in the interval of *QSd.sau-2SY-2D* were obtained (Supplementary Table 3a). In addition, based on the genome of wild emmer and CS v2.1, there were 30, 27 and 25 orthologs obtained for *QSd.sau-2SY-7A.2*, *QSd.sau-AM-5A.2*, and *QSd.sau-AM-7B*, respectively (Supplementary Tables 3b–d).

### Quantitative Trait Loci × Environment and Epistatic Interactions for Spike Density

There were 5 and 11 QTL for SD identified by QTL and environment interaction analysis in the two RIL populations (Supplementary Table 4), respectively. Four of them (*QSd.sau-2SY-7A.2, QSd.sau-AM-5A.2, QSd.sau-AM-7B*, and *QSd.sau-2SY-2D*) were identical to the three major and one stable QTL which were detected by single-environment analysis, suggesting they are stably expressed loci. In addition, ten pairs of QTL were detected by epistatic interaction analysis in two populations. However, all of them were identified in only a single environment and there were no interactions between QTL identified by single-environment analysis (Supplementary Table 5).

### Quantitative Trait Loci Validation

As *QSd.sau-2SY-7A.2* identified in the present study and *QSns.sau-2SY-7A* for SNS reported by Ding et al. (2021) correspond to the same interval, they may be regulated by the same locus. The SNS QTL has been verified in the 2CM population. Therefore, the phenotypic value of SD was calculated and verified in the present study based on the SNS and SL data from the 2CM population.

**FIGURE 5:**
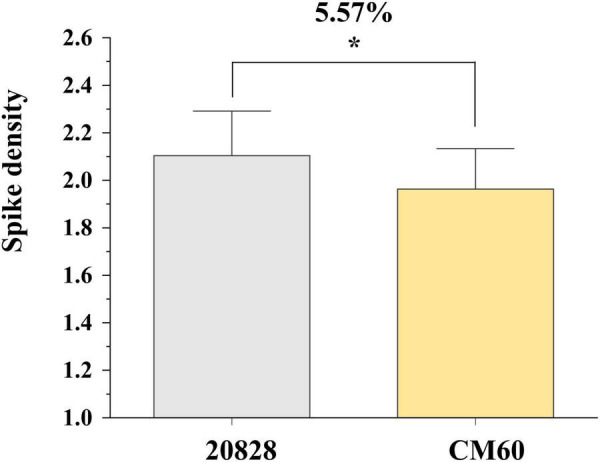
Effects of *QSd.sau-2SY-7A.2* in 20828 × CM60 (2CM) population. * Significance level at *P* < 0.05.

Student’s *t*-test was performed to compare phenotypes of lines homozygous for the alleles from 20828 with those from CM60. There were significant differences in phenotypic values between the two genotypes (*P* < 0.05) (Figure 5). The SD values of the lines homozygous for the allele from 20828 were obviously higher than those without the corresponding alleles. These results indicated that *QSd.sau-2SY-7A.2* should be a reliable and major SD locus.

### Effects of Major Quantitative Trait Loci on Spike Density in the 2SY and AM Populations

The interactions between *QSd.sau-2SY-2D* and *QSd.sau-2SY-7A.2* and between *QSd.sau-AM-5A.2* and *QSd.sau-AM-7B* were further analyzed, respectively.

**FIGURE 6:**
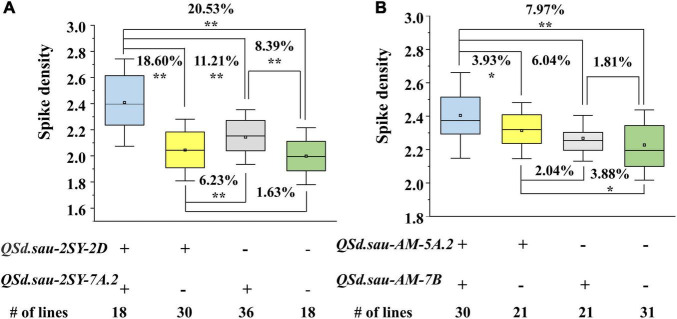
The aggregation effect of the major QTL for spike density (SD) in two RIL populations. **(A)** Effect of *QSd.sau-2SY-2D* and *QSd.sau-2SY-7A.2* for SD in the 2SY population; **(B)** Effect of *QSd.sau-AM-5A.2* and *QSd.sau-AM-7B* for SD in the AM population; + and − represent lines with and without the positive alleles of the corresponding QTL based on the flanking marker of the corresponding QTL, respectively; **Significant at *P* < 0.01, *Significant at *P* < 0.05.

Based on their flanking markers, the two populations were divided into four groups. In the 2SY population, compared to lines without any positive alleles of *QSd.sau-2SY-2D* or *QSd.sau-2SY-7A.2*, the SD of lines containing both the positive alleles of these two QTL was significantly increased by 20.53%. When the positive allele from *QSd.sau-2SY-2D* or *QSd.sau-2SY-7A.2* was expressed alone, SD increased by 1.63 and 8.39%, respectively (Figure 6A). In the AM population, *QSd.sau-AM-5A.2* and *QSd.sau-AM-7B* have a strong association with SD. Lines with a combination of positive alleles from *QSd.sau-AM-5A.2* and *QSd.sau-AM-7B* significantly increased SD by 7.97%, compared to those without any of the positive alleles. However, SD can only increase by 3.88 or 1.81% when the positive allele from *QSd.sau-AM-5A.2* or *QSd.sau-AM-7B* was present alone, respectively (Figure 6B).

### Effects of Major and Stable Quantitative Trait Loci on Yield-Related Traits

**FIGURE 7:**
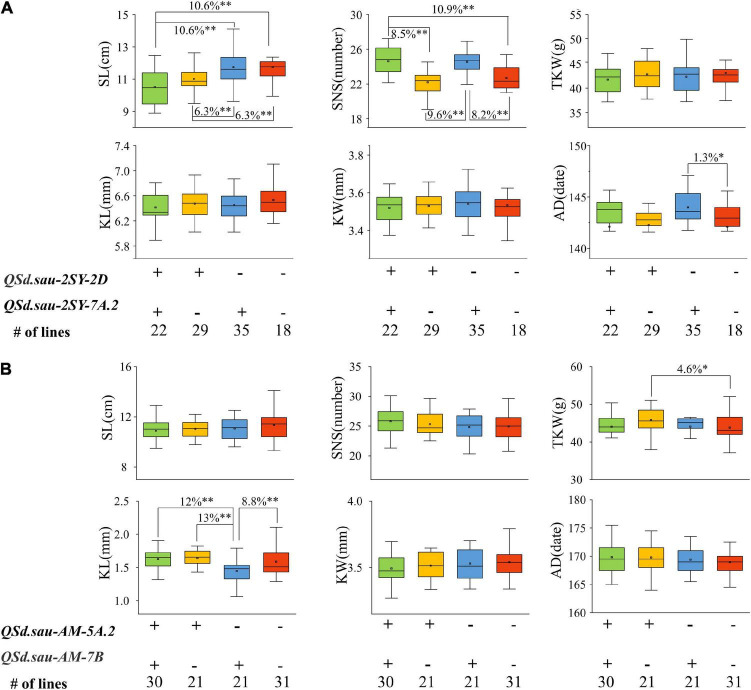
The effects of major quantitative trait loci (QTL) on yield-related traits in the 20828/SY95-71 (2SY) population **(A)** and AL /LM001 (AM) population **(B)**. SL, spike length; SNS, spikelet number per spike; TKW, thousand kernel weight; KL, kernels length; KW, kernels width; AD, anthesis date; + and − represent lines with and without the positive alleles of the target QTL based on the flanking markers the corresponding QTL, respectively. *Significance level at *P* < 0.05; ^**^Significance level at *P* < 0.01.

We further performed analysis of the effects of major and stable QTL for SD on other yield-related traits. In the 2SY population, as shown in Figure 7A, significant differences for SL, SNS, and AD existed among the different lines carrying various alleles. Specifically, SL of 22 lines possessing a combination of positive alleles from *QSd.sau-2SY-2D* and *QSd.sau-2SY-7A.2* were significantly lower (*P* < 0.01, 10.6%) than those carrying the positive allele from *QSd.sau-2SY-7A.2* only and those without *QSd.sau-2SY-2D* or *QSd.sau-2SY-7A.2*. Moreover, highly significant difference (*P* < 0.01) was detected between the lines carrying increased alleles from *QSd.sau-2SY-2D* and *QSd.sau-2SY-7A.2*. Compared with those without positive alleles from *QSd.sau-2SY-2D* or *QSd.sau-2SY-7A.2*, lines possessing that from *QSd.sau-2SY-2D* extremely and significantly (*P* < 0.01) reduced SL by 6.3%. For SNS, the phenotypic values of lines with a combination of *QSd.sau-2SY-2D* and *QSd.sau-2SY-7A.2* increased alleles were extremely and significantly higher than those with *QSd.sau-2SY-2D* increased allele or those without *QSd.sau-2SY-2D* or *QSd.sau-2SY-7A.2*, respectively. SNS of 29 lines possessing increased alleles from *QSd.sau-2SY-2D* was extremely and significantly (*P* < 0.01, 9.6%) lower than that of 35 lines from *QSd.sau-2SY-7A.2*. Additionally, compared with those without increased alleles from *QSd.sau-2SY-2D* or *QSd.sau-2SY-7A.2*, lines possessing alleles from *QSd.sau-2SY-7A.2* extremely and significantly (*P* < 0.01) increased SNS by 8.2%. In terms of AD, significant differences in phenotypic values were found only in lines carrying increased alleles from *QSd.sau-2SY-7A.2* and those none carrying *QSd.sau-2SY-7A.2* or *QSd.sau-2SY-2D* increased alleles.

In the AM population, for KL, compared with the lines with increased allele from *QSd.sau-AM-7B*, those from a combination of *QSd.sau-AM-5A.2* and *QSd.sau-AM-7B* extremely and significantly (*P* < 0.01) increased KL up to 12% (Figure 7B). Furthermore, the phenotype value of lines harboring *QSd.sau-AM-5A.2* increased alleles was significantly higher than those containing *QSd.sau-AM-7B* with difference being up to 13% (*P* < 0.01). The KL of 31 lines with positive allele from *QSd.sau-AM-7B* was significantly higher than those possessing the negative one. For TKW, compared with those without increased alleles from *QSd.sau-AM-5A.2* or *QSd.sau-AM-7B*, lines carrying alleles from *QSd.sau-AM-5A.2* extremely and significantly increased (*P* < 0.01, 8.2%). In addition to the significant differences described above, no differences were detected among lines with or without different positive alleles of the target QTL based on the flanking markers of the corresponding QTL. The above results indicated that QTL controlling SD significantly impacted SL, SNS, KL, AD, and TGW.

## Discussion

### The Possibility of Detection of Quantitative Trait Loci for Spike Density in the Mapping Populations Where No Significant Differences Are Present Between Their Parents

Based on the phenotypic analysis for 2SY and AM populations, there were no significant differences for SD between parents in multiple environments. Similarly, SNS between the parental lines exhibited no difference as well in AM population (Mo et al., 2021). However, we observed an approximately normal distribution and transgressive segregation in the two RIL populations, which conforms to the characteristics of quantitative traits. Above all, 18 QTL for SD were identified in this study and some of these major or stably expressed QTL have been proved to be reliable given their co-localization with other loci previously reported.

In fact, this phenomenon exists in many QTL mapping studies (Zhou et al., 2017; Liu et al., 2019). Previous studies have shown that the phenotype of a trait is usually established by an interaction of several genes (Liu et al., 2019), such as reciprocal inhibition between genes causing the lack of a corresponding phenotype for these genes. However, through hybridization between two given genotypes, genetic recombination makes it possible for an offspring to carry a locus independent of other inhibited loci, and thus the corresponding phenotype can be expressed. Therefore, even if the parental phenotypes are not significantly different, it is possible to identify major QTL for a given traits in a RIL population.

### Major Quantitative Trait Loci for Spike Density

In this study, four QTL *QSd.sau-2SY-2D.3*, *QSd.sau-2SY-7A*, *QSd.sau-AM-5A.2*, and *QSd.sau-AM-7B* were identified on chromosomes 2D, 5A, 7A, and 7B, and they showed high PVE and were expressed in multiple environments. According to genomes of CS, wild emmer, and *A. tauschii*, these QTL were anchored in the corresponding reference genome intervals, respectively (Figure 3).

Many QTL controlling SD have been identified by genetic analysis. To further determine whether these QTL in this study are novel loci, we obtained the physical locations of previously reported QTL and genes associated with SD (Supplementary Table 6).

In the CS genome, the *QSd.sicau-2D.3* identified by Liu et al. (2019) overlapped with *QSd.sau-2SY-2D* between 605.12 and 609.88 Mb on chromosome 2DL (Figure 3A); *qSc-7A* (Fan et al., 2019), explaining 4.87–17.22% of variation in SD, was located between 679.70 and 679.92 Mb, and *QSd.sau-2SY-7A.2* was mapped to between 678.47 and 682.29 Mb (Figure 3B), indicating that they may be allelic variants. In addition, comparing with the physical maps of QTL for other yield-related traits such as SNS (Ding et al., 2021), KL, KW, kernel thickness (KT), TKW, kernel length–width ratio (LWR), and kernel size (KS) (Qu et al., 2021), none of the physical regions of QTL for SD in the present study overlapped with these yield-related loci.

*QSd.sau-AM-5A.2* and *QSd.sau-AM-7B* were identified in the AM population. Specifically, *QSd.sau-AM-5A.2*, located between 618.87 and 621.62 Mb (Figure 3C), was determined to be distinct from *Q* (651.82 Mb) and *Vrn-A1* (589.27 Mb). There were no overlapping regions with other QTL or genes for SD reported on 5AL comparing with the previous studies (Supplementary Table 6). We also performed QTL analysis for KNS and KNL in the AM population (Supplementary Table 7), and results indicated that two minor QTL *QKns.sau-AM-5A* and *QKnl.sau-AM-5A* were both located in the physical interval 619.18–622.72 and 613.60–617.54 Mb on wild emmer and CS genome, respectively. These two loci were close to the interval of *QSd.sau-AM-5A.2*, suggesting they may be controlled by a pleiotropic locus.

*QSd.sau-AM-7B*, located between 684.82 and 687.74 Mb (Figure 3D), was determined to be close to *Sd.sicau-7B.1* (688.53–689.86 Mb) (Liu et al., 2019), suggesting they may be allelic. Thus, comparisons showed that *QSd.sau-AM-5A.2* may be a novel and major QTL controlling SD.

### Comparison of *QSd.sau-AM-5A.2* to Other Loci for Spike Length and Spikelet Number Per Spike

Spike density and spike length as well as SNS are tightly correlated traits and many QTL for SNS and SL also have pleiotropic effects on SD, and co-location of QTL related to these three traits has been reported in many studies. For example, QTL conferring SNS, SL, KNS, and TKW were co-located at *Xgwm126* – *Xgwm291* (672.92–700.49 Mb; Wang et al., 2011); *QHd.sau-5A*, *QAn.sau-5A*, *QPht.sau-5A*, *QSl.sau-5A1*, and *QSd.sau-5A1* were co-located at *wPt-9094–wPt-9513* (435.00–536.98 Mb; Luo et al., 2016). Compared with kernel size-related QTL in the AM population, no QTL for such as KL and KW were detected on chromosome 5A (Zhou et al., 2021). Additionally, *QSd.sau-AM-5A.2* (624.11–626.14 Mb on wild emmer genome) in the present study and *QSns.sau-AM-5A* (557.72–571.41 Mb; Mo et al., 2021) are not co-located in the same region. A great deal of QTL or genes related to yield traits existed on chromosome 5A. To identify whether *QSd.sau-AM-5A.2* is co-located with QTL or genes for SNS or SL reported previously, based on the comparison of QTL for SNS on chromosome 5A with previous studies (Mo et al., 2021), we reviewed the recently published articles related to SL and SNS (Supplementary Table 8). Results showed that many SL or SNS related loci located on chromosome 5AL, but most of the reported QTL or genes were at least tens of Mb away from *QSd.sau-AM-5A.2* detected in this study, such as *QSl.cib-5A* (516.60–521.27 Mb; Ji et al., 2021), *QTgw.cau.5A_140-142* (698.00–705.40 Mb) and *QSsi.cau.5A_91* (586.61–589.22 Mb; Wang et al., 2021), *QSl.wa-5AL.e1*/*QSl.wa-5AL.e2*/*QSns. wa-5AL.e1*/*QSns.wa-5AL.e2*/*QSns.wa-5AL.e3*/ *QGns.wa-5AL.e2*/ *QTgw.wa-5AL.e2*/*QTgw.wa-5AL.e3* (672.92–700.49 Mb; Wang et al., 2011). However, Cui et al. (2012) reported that a major locus *QSl.WY.5A.1* for SL was marked with *Xcfa2163.2* and *Xcwm216*, and this locus was tightly closed to *QSd.sau-AM-5A.2* in the physical interval (Supplementary Table 8), indicating pleiotropic effects might exist between them.

### Pyramiding Analysis of Major Quantitative Trait Loci for Spike Density

Previous studies have found that integrating multiple favorable QTL into the same genetic background can significantly optimize plant traits (Fan et al., 2019; Li et al., 2021). This pyramiding effect of multiple loci is an effective means for the improvement of modern wheat varieties.

In the present study, three major QTL and one stable QTL were detected in the two mapping populations. The pyramiding effect analysis was used to further verify the role of those QTL and analyze the relationships among them. The lines carrying a combination of positive alleles of two given QTL showed significantly greater SD than those from other lines. This indicated an additive effect between these two QTL increasing SD. Through the accumulation of elite genes, an improved SD phenotype can be constructed.

### Phenotypic Correlations Between Spike Density and Other Agronomically Important Traits

Spike density, obtained by dividing SNS by SL, is an important factor in cultivating high-yield wheat. Therefore, theoretically, SD should be positively correlated with SNS and negatively correlated with SL. In the present study, the Pearson’s correlation analysis showed that SD was indeed negatively correlated with KL and SL and positively correlated with SNS. Moreover, the correlation between SD with TKW was positive in the 2SY population.

Most of the loci controlling spike traits of wheat were observed to be closely linked in previous studies. Major loci for SNS and SD in wheat identified by Fan et al. (2019) were clustered within the same confidence interval on chromosome 7AL. Similarly, Zhai et al. (2016) detected multiple spike QTL in two winter variety populations, among which QTL associated with SD and SL were included in the two genomic regions on chromosomes 2D and 5A, respectively. These studies revealed the potential for pleiotropism of corresponding traits and also revealed the genetic correlations among SD, SL, and SNS. In addition, higher SD may result in shorter kernels and smaller TKW, being consistent with the study reported by Qu et al. (2021). Thus, it is essential to determine relationships among different characters to accelerate breeding process.

### Analysis of Candidate Genes

Candidate genes were analyzed in the regions with major and stable QTL. Further analysis indicated that some genes are related to the regulation of plant growth, and they may affect the formation of the spike in wheat.

For example, *TraesCS2D03G1128600* encodes an AP2-like transcription factor that plays a crucial role in flower development. Previous studies have reported that AP2 was involved in growth and development of the floral organs and seed development (Kunst et al., 1989; Schultz and Haughn, 1993; Okamuro et al., 1997). In the physical interval of *QSd.sau-2SY-7A.2*, we found that *TraesCS7A03G1166400* encoding the UNUSUAL FLORAL ORGANS (UFO) protein was involved in floral meristem development and was a key regulatory factor in floral bud differentiation (Levin and Meyerowitz, 1995). *Traescs7A03G1173200* encodes a receptor-like protein kinase that regulates the expression of floral meristem formation factors and promotes cell proliferation to control flower development and organ growth in *Arabidopsis thaliana* (Shpak et al., 2004).

The candidate gene *TRIDC5AG062530* on chromosome 5A controls flowering time by encoding a FRIGIDA-like protein, leading to a late flowering phenotype in plants (Yang et al., 2012). *TRIDC7BG064700*, a candidate gene for *QSd.sau-AM-7B*, encodes a DA2 protein. Xia et al. (2013) found that DA2 can regulate seed size by restricting cell proliferation in the integument.

Furthermore, it is noteworthy that five genes identified in this study (*TraesCS7A03G1167800, TraesCS7A03G1169000, TraesCS7A03G1169300, TRIDC5AG062440*, and *TRIDC5AG062450*) encode cortical cell-delineating proteins. Previous research has revealed that cortical cell-delineating proteins affect plant morphological development by regulating cell division and expansion (Fu et al., 2009). Therefore, these candidate genes may be of vital significance for understanding the genetic mechanism of spike development in wheat. They may also provide clues for further fine mapping of major QTL.

## Conclusion

In this study, three major QTL and a stable one, located on chromosomes 5A, 7A, 7B, and 2D, were identified in two independent wheat RIL populations. Based on genetic analysis, *QSd.sau-2SY-2D, QSd.sau-2SY-7A.2*, and *QSd.sau-AM-7B* were found to overlap with reported SD loci. However, *QSd.sau-AM-5A.2* identified in the tetraploid wheat RIL population may be a new QTL. Furthermore, the correlations between SD and other agronomic traits, and the candidate genes related to spike development in the corresponding loci were analyzed and discussed, laying a foundation for subsequent fine mapping.

## Data Availability Statement

The original contributions presented in the study are included in the article/Supplementary Material, further inquiries can be directed to the corresponding authors.

## Author Contributions

JY and HL performed the entire study and drafted this manuscript. SW did phenotype measurement and data analysis. WL and LG did field work and data analysis. QJ, GC, PQ, and YP helped with data collection and analysis. HT, YM, and MD helped with field work and phenotype measurement. LT, AH, YW, and YZ discussed results. XL guided the study and revised the manuscript. JM designed the experiments, guided the entire study, participated in data analysis, wrote, and extensively revised this manuscript. All authors participated in the research and approved the final manuscript.

## Conflict of Interest

The authors declare that the research was conducted in the absence of any commercial or financial relationships that could be construed as a potential conflict of interest.

## Publisher’s Note

All claims expressed in this article are solely those of the authors and do not necessarily represent those of their affiliated organizations, or those of the publisher, the editors and the reviewers. Any product that may be evaluated in this article, or claim that may be made by its manufacturer, is not guaranteed or endorsed by the publisher.

## Footnotes

1
https://www.originlab.com/

2
http://202.194.139.32/

3
http://www.uniprot.org/
